# Spatial relationships among public places frequented by families plagued by methicillin-resistant *Staphylococcus aureus*

**DOI:** 10.1186/s13104-018-3797-4

**Published:** 2018-10-01

**Authors:** Katelyn L. Parrish, Patrick G. Hogan, Arvon A. Clemons, Stephanie A. Fritz

**Affiliations:** Department of Pediatrics, Washington University School of Medicine, 660 S. Euclid Avenue, CB 8116, St. Louis, MO 63110 USA

**Keywords:** *Staphylococcus aureus*, Community-associated methicillin-resistant *Staphylococcus aureus* (CA-MRSA), Geographic information systems (GIS)

## Abstract

**Objective:**

To understand factors associated with community-associated methicillin-resistant *Staphylococcus aureus* (CA-MRSA) acquisition and infection, we mapped public places (including personal service establishments, fitness centers, pools, schools, and daycares) visited by members of households affected by CA-MRSA skin and soft tissue infection.

**Results:**

From January 2012 to October 2015, households of children with CA-MRSA SSTI in metropolitan St. Louis were enrolled in the HOME: Household Observation of MRSA in the Environment study. Addresses of public places visited within 3 months of enrollment were reported by 671 participants and were analyzed using a geographic information system (GIS). The Nearest Neighbor Tool in ArcGIS assessed clustering of public places within the study region. Public places were significantly clustered within the study area compared to the expected distance between locations (p < 0.001). Additionally, one-third (48/150) of participating households visited at least one public place in common with other households. No significant relationship between participants visiting the public places within 3 months of enrollment and subsequent colonization or SSTI were found. Understanding community behavior is critical to informing public health initiatives to reduce the prevalence of CA-MRSA infections.

## Introduction

*Staphylococcus aureus* is a commensal organism, colonizing approximately one-third of the population [1]. *S. aureus* is also a successful pathogen, causing a spectrum of infections from skin and soft tissue infection (SSTI) to severe, life-threatening infections [2]. Community-associated methicillin-resistant *S. aureus* (CA-MRSA) has emerged as the leading cause of SSTI in otherwise healthy people [3]. CA-MRSA is transmitted by direct skin-to-skin contact or contact with a contaminated surface, and can survive on surfaces for months [4]. Currently, there is equipoise surrounding the most effective measures to combat the transmission of *S. aureus* in the community.

*Staphylococcus aureus* contamination has been documented in public places such as locker rooms, exercise facilities and daycare centers [5, 6]. Understanding community transmission dynamics which contribute to *S. aureus* acquisition is essential. There may be an underlying spatial relationship in populations where CA-MRSA prevalence is high. Utilizing tools such as geographic information systems (GIS) allows researchers to reveal and understand disease trends within populations that may not be visible using other methods. Previous studies have investigated the role of geography as a risk factor for developing MRSA SSTI [7]; to our knowledge, no study has mapped public places visited by pediatric patients and their household contacts to elucidate factors contributing to MRSA acquisition and transmission.

We mapped public places visited by pediatric index patients with acute MRSA SSTI and their household contacts—enrolled in a larger study of household MRSA transmission—in order to determine whether: (1) public places visited by participants were significantly clustered within the study area; (2) household members from different households affected by CA-MRSA were visiting the same public places; and (3) public places visited by household members in the 3 months prior to index patient acute MRSA SSTI were associated with *S. aureus* colonization or incidence of SSTI.

## Main text

### Methods

Recruitment for the HOME: Household Observation of MRSA in the Environment study took place between January 2012 and October 2015, as previously described, following approval from the Washington University Institutional Review Board [8]. Otherwise healthy pediatric patients presenting with a recent culture-confirmed CA-MRSA SSTI (i.e., index patients) living in metropolitan St. Louis were enrolled along with their household contacts. Participants were recruited from St. Louis Children’s Hospital (SLCH), Cardinal Glennon Children’s Hospital, and Washington University Pediatric and Adolescent Ambulatory Research Consortium (WU PAARC)-affiliated community practices. All participants provided written informed consent/assent and parents/guardians provided consent on behalf of minors.

At enrollment the investigative team swabbed participants in the anterior nares, axillae, and inguinal folds (Eswab, Becton–Dickinson [BD], Franklin Lakes, NJ) to determine *S. aureus* colonization status. *S. aureus* isolation, identification, and antibiotic susceptibility testing methods have been previously reported [8]. A survey was administered regarding potential factors associated with *S. aureus* colonization and infection including medical history, hygiene practices, and activities. Questions about activities assessed whether participants had been to public places such as a hair salon, day spa, nail salon, tanning bed, fitness gym, used a public locker room, used a public shower, swam in a public pool, and/or used a public sauna/hot tub within 3 months of enrollment. Additionally, information was collected regarding daycare, school, or before/after school program attendance. The name/location of all non-residential facilities were recorded.

Addresses for public places were compiled from participant survey responses. Locations were excluded from the analysis if the description was too vague to find a corresponding address (e.g., participants provided nonspecific names of franchises with multiple nearby locations). Addresses were assigned geographical coordinates using ArcGIS version 10.4.1 (Esri, Redlands, California) and analyzed using the Average Nearest Neighbor tool, which provides a ratio of the observed mean distance and the expected mean distance between locations (Nearest Neighbor Index) [9]. An index of < 1 indicates that the locations exhibit clustering within the study area compared to the distance expected by chance alone; p-values < 0.05 were considered significant. The Nearest Neighbor Index was calculated for daycare centers, schools, and before/after school programs for children < 18 years old (N = 360). Clustering of all other public places was analyzed for the entire cohort (N = 671). Additionally, address frequency analysis and Nearest Neighbor analysis were performed at the household level to determine whether study participants from more than one household visited the same public places, and if these locations were spatially related. Sub-analysis was conducted to determine the median driving distance from participating households’ home addresses to the most commonly visited public places. Logistic regression was performed in SPSS version 25 for Windows (IBM SPSS, Chicago, IL) to determine whether visiting any public places at least once a month in the 3 months prior to enrollment was associated with SSTI and/or *S. aureus* colonization.

### Results

One-hundred fifty households comprised of 671 participants were enrolled. Participants resided in metropolitan St. Louis, Missouri, extending into western Illinois, spanning 120 miles. Median household distance from SLCH was 17.3 miles (range 0.9–76.0 miles). Forty-seven percent of participants were male and the median age among participants was 15.0 years (0.1-82.3). Participants mostly identified as Caucasian (69%), while 27% were African-American; 5% of participants identified as Latino/Hispanic. Forty-one percent of participants were colonized with *S. aureus* at enrollment: 21% with MRSA, 17% with methicillin-susceptible *S. aureus* (MSSA), and 3% with both MRSA and MSSA at different anatomic sites.

**Fig. 1:**
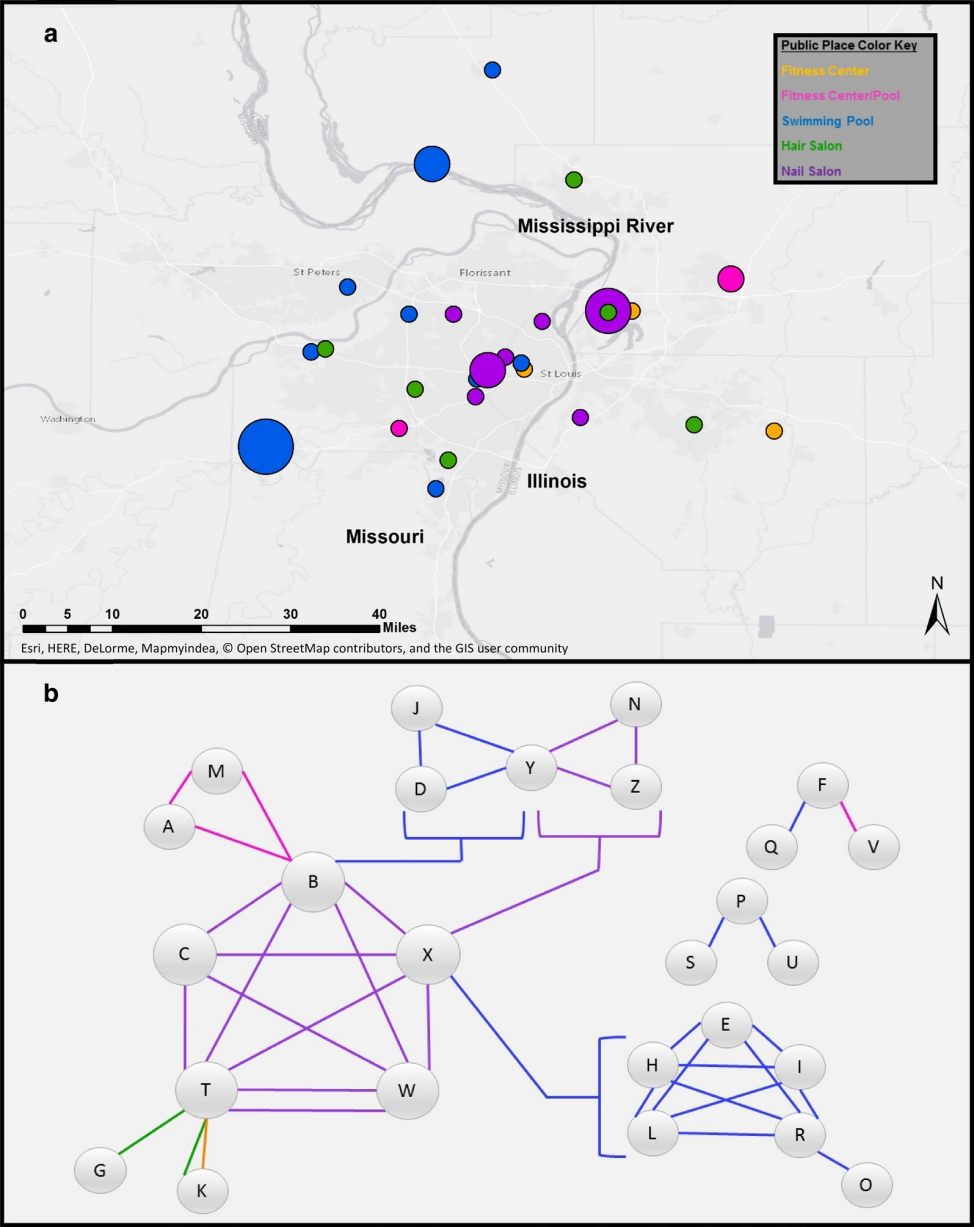
**a** Frequency of use of the 28 most common public places visited by multiple households. The spatial relationship among all public places visited by more than one household as reported by participants in the study is depicted. Category of public place is indicated by the color of the circle. Size of the circles on the map reflects the number of households that had at least one member visit the location (range 2–6 households). Base map retrieved via ESRI ArcGIS from OpenStreetMap. **b** Display of public places visited by multiple households. The spatial relationship between select households visiting the same public places as reported by study participants is depicted. Circles represent households (each household ID represented by a capital letter). A line connecting two circles indicates that at least one member of the household ID in each circle visited the same public place. Category of public place is indicated by the color of the line. Brackets indicate that a member of the household visited the same location as a member of each household enclosed within the brackets (e.g., household B attended the same swimming pool as households D, J, and Y). Households T and W both visited two of the same nail salons (indicated by two purple lines between these households). Forty-eight households visited public places that were also visited by at least one other study household. Twenty-six households are included in the figure as they are the households with the most public locations in common

Among daycare centers, schools, and before/after school programs, the Nearest Neighbor Index was 0.109 (p < 0.001), indicating clustering. For hair salons, day spas, nail salons, tanning beds, fitness gyms/exercise facilities, public showers, public locker rooms, public pools, and public saunas/hot tubs/Jacuzzis, the Nearest Neighbor Index was 0.129 (p < 0.001), also indicating clustering. Clustering within the most frequently visited categories of public places (Fig. 1a) (fitness gyms, hair salons, nail salons, and public pools) was also significant (p < 0.001 for each analysis). Median driving distance between the locations visited by more than one household and the home address of the households that visited them was 12.5 miles (range 0.8–58.5 miles), suggesting that these frequently visited locations were not shared among households based solely on geographic proximity to their homes.

Address frequency analysis revealed that 48 of 150 households (32%) had a member who visited at least one of the same public places as a member of another participating household. Five households (3%) had at least one member that visited a public place that was also visited by a member of two or more participating households. For example, a member(s) of Household B went to three different locations within the three months prior to study enrollment—a nail salon, a pool, and a fitness center—that had been visited by members of four, three and two other study households, respectively (Fig. 1b). Members of Household T visited the same locations as study participants from six other households, twice overlapping with Household K. Members of Household X visited three public locations that a member(s) of 12 other households also visited. There were no independent associations between visiting any of the public places at least one time per month in the 3 months prior to enrollment and *S. aureus* colonization or SSTI (Table 1).

**Table 1:** Odds of SSTI or *S. aureus* colonization given locations visited in the 3 months prior to household MRSA study enrollment

Location and frequency	SSTI incidence				*S. aureus* colonization			
	Participants with SSTI N = 216 (%)	Participants without SSTI N = 455 (%)	OR (95% CI)	aOR^a^ (95% CI)	Participants with *S. aureus* colonization N = 275 (%)	Participants without *S. aureus* colonization N = 396 (%)	OR (95% CI)	aOR^b^ (95% CI)
Hair salon			0.78 (0.53, 1.14)	0.98 (0.64, 1.50)			1.05 (0.74, 1.50)	1.04 (0.72, 1.50)
≥ 1×/month	47 (22)	120 (26)			70 (25)	97 (24)		
< 1×/month	169 (78)	335 (74)			205 (75)	299 (76)		
Day spa			0.84 (0.16, 4.37)	1.81 (0.32, 10.2)			0.24 (0.28, 1.98)	0.27 (0.03, 2.28)
≥ 1×/month	2 (1)	5 (1)			1 (0.4)	6 (2)		
< 1×/month	214 (99)	450 (99)			274 (99.6)	390 (98)		
Nail salon			0.48 (0.24, 0.94)	0.86 (0.40, 1.81)			1.23 (0.71, 2.12)	1.47 (0.82, 2.64)
≥ 1×/month	11 (5)	46 (10)			26 (9)	31 (8)		
< 1×/month	205 (95)	409 (90)			249 (91)	365 (92)		
Tanning bed			0.90 (0.23, 3.52)	1.42 (0.34, 5.9)			1.45 (0.42, 5.05)	1.67 (0.47, 5.90)
≥ 1×/month	3 (1)	7 (2)			5 (2)	5 (1)		
< 1×/month	213 (99)	448 (98)			270 (98)	391 (99)		
Fitness center			0.96 (0.63, 1.46)	1.40 (0.88, 2.23)			0.76 (0.50, 1.14)	0.78 (0.52, 1.18)
≥ 1×/month	38 (18)	83 (18)			43 (15)	78 (20)		
< 1×/month	178 (82)	372 (82)			232 (85)	318 (80)		
Locker room			1.01 (0.65, 1.57)	1.25 (0.78, 2.00)			0.99 (0.65, 1.50)	1.01 (0.66, 1.54)
≥ 1×/month	35 (16)	73 (16)			44 (16)	64 (16)		
< 1×/month	181 (84)	382 (84)			231 (84)	332 (84)		
Public shower			0.96 (0.44, 2.05)	1.67 (0.71, 3.90)			0.99 (0.48, 2.02)	1.07 (0.51, 2.22)
≥ 1×/month	10 (4)	22 (5)			13 (5)	19 (5)		
< 1×/month	206 (96)	433 (95)			262 (95)	377 (95)		
Public pool			1.23 (0.82, 1.83)	1.07 (0.70, 1.66)			1.01 (0.69, 1.49)	1.01 (0.68, 1.50)
≥ 1×/month	47 (22)	84 (18)			54 (20)	77 (19)		
< 1×/month	169 (78)	371 (82)			221 (80)	319 (81)		
Public Jacuzzi			0.94 (0.29, 3.07)	1.85 (0.51, 6.76)			1.24 (0.41, 3.73)	1.35 (0.45, 4.11)
≥ 1×/month	4 (2)	9 (2)			6 (2)	7 (2)		
< 1×/month	212 (98)	446 (98)			269 (98)	389 (98)		

Binary logistic regression performed using SPSS version 25 for Windows (IBM SPSS, Chicago, IL)

*SSTI* skin and soft tissue infection, *OR* odds ratio, *aOR* adjusted odds ratio

^a^SSTI incidence adjusted for age, gender, race, ethnicity, and *S. aureus* colonization status

^b^*S. aureus* colonization adjusted for age, gender, race, and ethnicity

### Discussion

Residential addresses have been shown to be a risk factor for MRSA infection [7, 10]. In order to devise interventions to stop the spread of *S. aureus*, it is important to understand community reservoirs for transmission. There is a dearth of information regarding visiting public places and risk of CA-MRSA infection. In this study, we demonstrated that, overall, public places visited by members of households affected by CA-MRSA infections are clustered within the study radius, meaning that there is a significant, non-random spatial relationship observed among them. Additionally, many study households were visiting the same public places, suggesting that those locations may be related to CA-MRSA transmission. We demonstrated that approximately one-third of the households in our study have been patrons of the same nail salons, hair salons, fitness centers, and swimming pools in the 3 months prior to enrollment in the study.

Previously published studies have shown a significant, positive relationship between utilizing personal care service establishments and SSTI [11]. Nail salons and hair salons are places where, if present, suboptimal sanitation and hygiene practices, such as the improper cleaning of tools (e.g. nail trimmers, towels, hairbrushes) could propagate MRSA on items used for multiple customers, leading to transmission. While nail salons have been established as a reservoir for infectious agents, such as *Mycobacteria* [11], there was no similar association in the present study between having been to locations named by participants in the 3 months prior to enrollment and having had an SSTI.

In the present study, participants visited many of the same public places (e.g., hair and nail salons and swimming pools) despite distance from their homes, which demonstrates the community-wide nature of the CA-MRSA threat. Further studies assessing the temporal relationship between CA-MRSA and public locations, as well as other community reservoirs, are necessary to understand community behavior, inform the development of interventions, and prevent transmission.

## Limitations

The cross-sectional nature of the study limits our ability to establish temporality among the study results. Additionally, study data were self-reported, thus there is the possibility of information bias, though it is likely nondifferential. Households were selected from a limited geographic region, and results may not be generalizable to other locations.

## Abbreviations

CA-MRSA: community-associated methicillin-resistant *Staphylococcus aureus*
GIS: geographic information system
MSSA: methicillin-susceptible *Staphylococcus aureus*
MRSA: methicillin-resistant *Staphylococcus aureus*
SLCH: Saint Louis Children’s Hospital
SSTI: skin and soft tissue infection
WU PAARC: Washington University Pediatric and Adolescent Ambulatory Research Consortium
